# Modified Anchor Shaped Post Core Design for Primary Anterior Teeth

**DOI:** 10.1155/2014/306910

**Published:** 2014-10-14

**Authors:** R. Rajesh, Kusai Baroudi, K. Bala Kasi Reddy, B. H. Praveen, V. Sumanth Kumar, S. Amit

**Affiliations:** ^1^Department of Paediatric and Preventive Dentistry, KLR'S Lenora Institute of Dental Sciences, Rajanagaram, Rajahmundry, Andhra Pradesh 533294, India; ^2^Department of Restorative Dental Sciences, Al-Farabi College, Riyadh 11691, Saudi Arabia; ^3^Department of Conservative Dentistry and Endodontics, MNR Dental College, Sangareddy, Telangana 502294, India; ^4^Department of Preventive and Community Dentistry, Kamineni Institute of Dental Sciences, Narketpally, Telangana 508254, India; ^5^Department of Paediatric and Preventive Dentistry, SVS Institute of Dental Sciences, Mahabubnagar, Telangana 509 001, India

## Abstract

Restoring severely damaged primary anterior teeth is challenging to pedodontist. Many materials are tried as a post core but each one of them has its own drawbacks. This a case report describing a technique to restore severely damaged primary anterior teeth with a modified anchor shaped post. This technique is not only simple and inexpensive but also produces better retention.

## 1. Introduction

Dental caries is a predominant cause for tooth loss. Over the past decade, its occurrence has decreased in developed countries [1]. Early child hood caries (ECC) is a rapidly developing and progressing type of dental caries occurring initially in the cervical third of the maxillary incisors and leading to the destruction of the crown completely. ECC primarily affects the maxillary primary incisors immediately after the eruption of teeth and infects other primary teeth quickly, causing early tooth loss, reduced masticatory efficiency, loss of vertical dimension, tongue thrusting, speech problems, malocclusion, space loss, and psychological problems [2].

In the last few decades of the advent of new materials like strip crowns, polycarbonate crowns, veneered stainless steel crowns, and art glass crowns, carious teeth with sufficient tooth structure are being restored esthetically and effectively [3–7]. This has led to a gradual shift from extraction to nonextraction treatment modalities [8, 9].

But these materials fail to withstand occlusal forces in severely damaged teeth with loss of crown structure. Hence post and core systems were introduced to provide additional support to the restorations. Some of the post and core systems being used are modified Omega shaped orthodontic wires, biological posts, fiber core posts, and glass reinforced fiber composites (GFRC) [10–12].

Fiber core posts and GFRC showed promising results but are expensive [13–16]. Lack of availability of tooth banks and lack of secure methods of sterilization and storage to ensure the safety of teeth are some of the disadvantages of biological post [17, 18]. Omega shaped orthodontic wires, on the other hand, are inexpensive but lack retention [15].

Hence we present an alternative approach to the restoration of severely damaged maxillary primary incisors using a modified anchor shaped orthodontic wire.

## 2. Case Report

**Figure 1:**
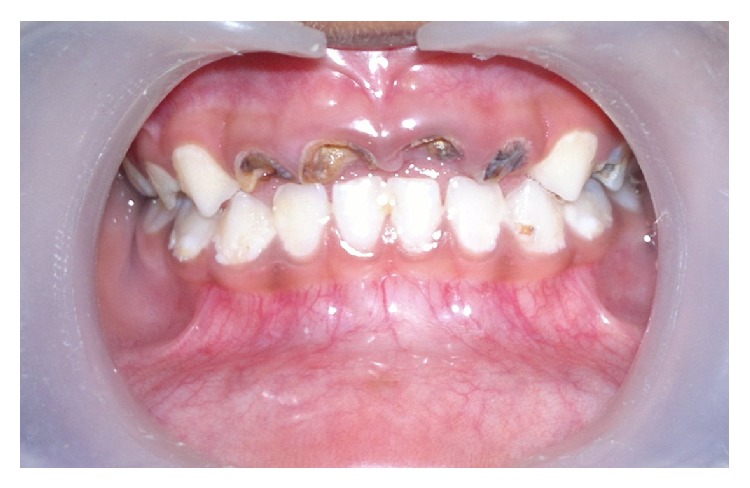
Preoperative clinical picture showing grossly decayed teeth 51, 52, 61, and 62.

**Figure 2:**
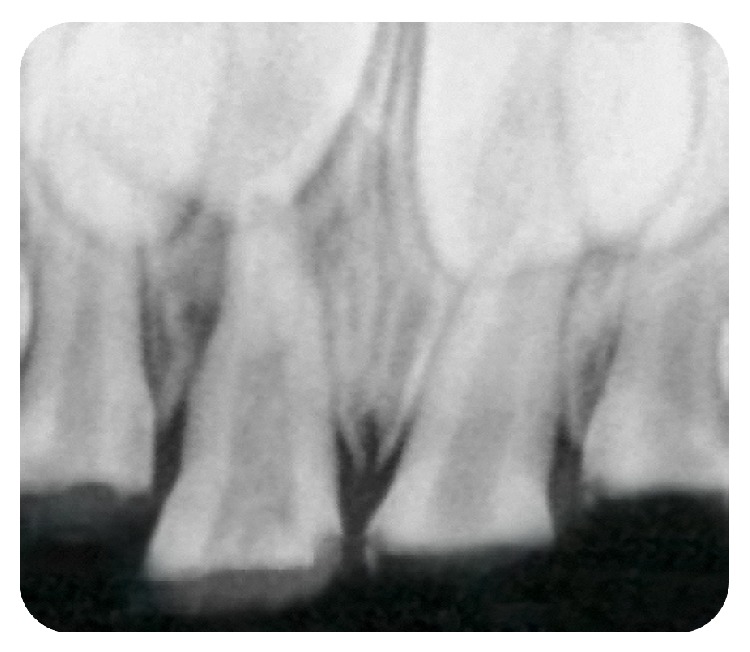
Preoperative radiograph showing teeth 51, 52, 61, and 62.

A 4-year-old female patient accompanied by her parents reported to our private dental clinic, with a chief complaint of decayed upper front teeth. Intraoral examination revealed a complete set of deciduous teeth. Clinically, 51, 52, 54, 61, 62, 64, 65, 73, 74, 75, 84, and 85 were found to be carious with loss of crown structure (Figure 1). Intraoral periapical radiograph shows the involvement of pulp in relation to 51, 52, 61, and 62 (Figure 2).

**Figure 3:**
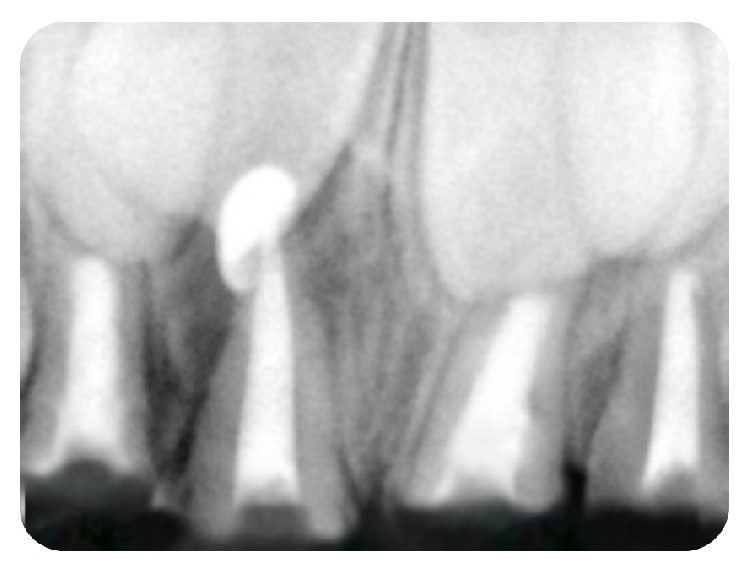
Postobturation radiograph in relation to 51, 52, 61, and 62.

The treatment goal was to remove the infected pulp and restore function and esthetics by recreating the normal architecture. Pulpectomy was performed on 51, 52, 61, and 62 under local anaesthesia. Obturation was done with calcium hydroxide (Metabiomed, Metapex) after debridement of the canal (Figure 3). About 2-3 mm of calcium hydroxide was removed from each canal and glass ionomer cement was placed over it (about 1 mm thickness). As it is difficult for the remaining tooth structure to bear the occlusal forces without any support, post and core restorations were advocated. The root resorption of the primary teeth is a key factor in the selection of the type of post and core. The post and core should not extend beyond 3-4 mm depth of root canal, as it may obstruct the normal path of eruption of the teeth. In this patient anchor shaped design of post and core was used.

## 3. Steps of Fabrication

**Figure 4:**
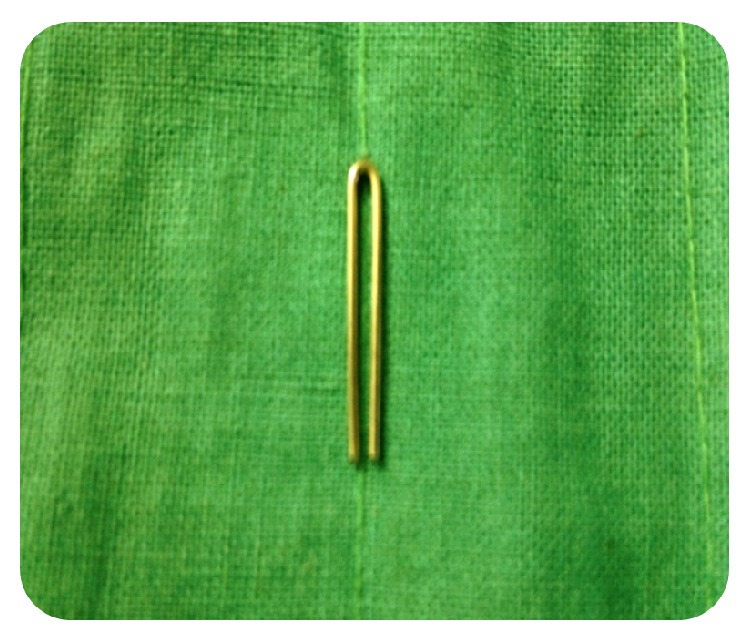
Fabrication of post and core step 1: 1.5 inch 19 gauge wire is bent to an inverted u shape.

**Figure 5:**
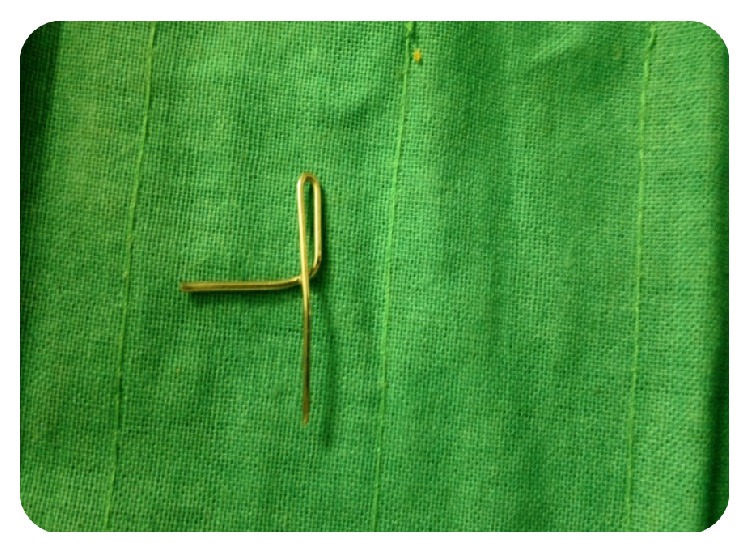
Fabrication of post and core: one of the arms' is bent downwards and turned to the opposite side.

**Figure 6:**
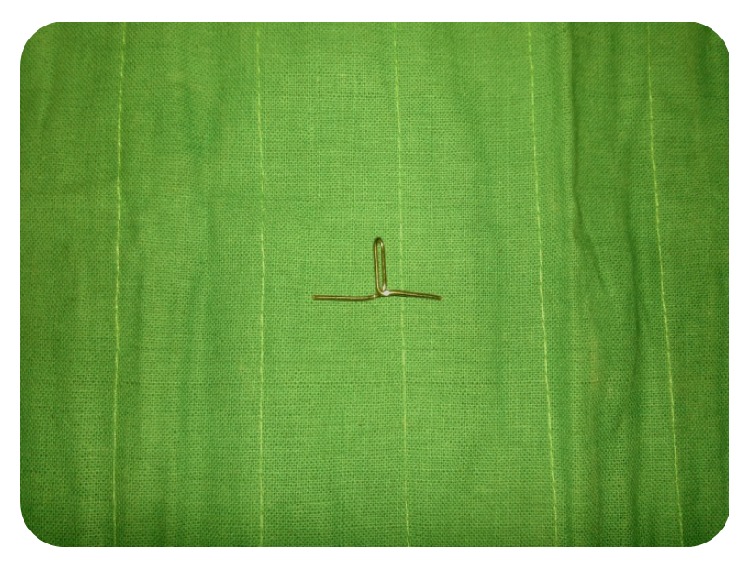
Fabrication of post core: procedure in Figure 5 repeated with the other arm.

**Figure 7:**
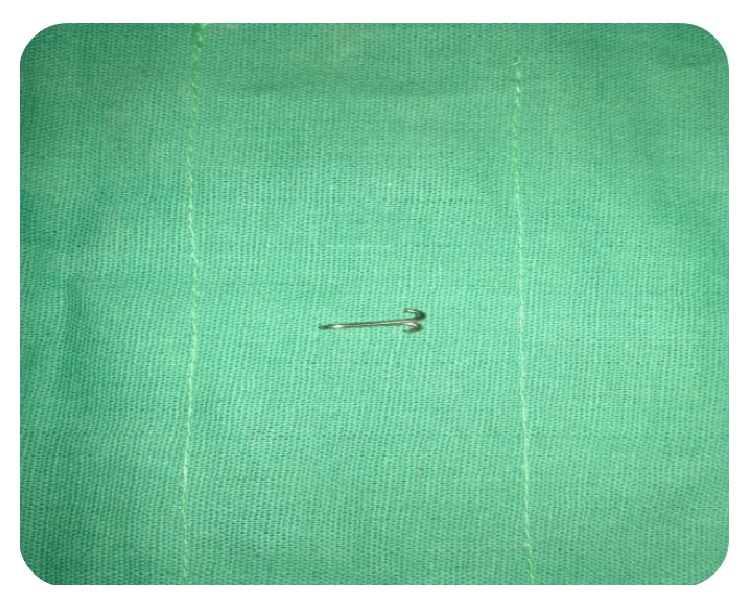
Fabrication of post core: bend the free end upwards and cut the excess to form an anchor shape.

A 19-gauge orthodontic wire, 1.5 inch in length, is bent using a universal plier as shown in Figure 4. Bend one of the arms downwards and turn it to the opposite side (Figure 5). Repeat the same procedure for the other arm (Figure 6). Bend the free end of the arms towards the curved end (Figure 7). Cut the excess wire as required. This gives an anchor shape to the retainer. By compressing the curved end, the free end opens up to adapt to the walls of the root thereby giving extra mechanical retention. Excess compression is not advised as it may cause root fracture.

**Figure 8:**
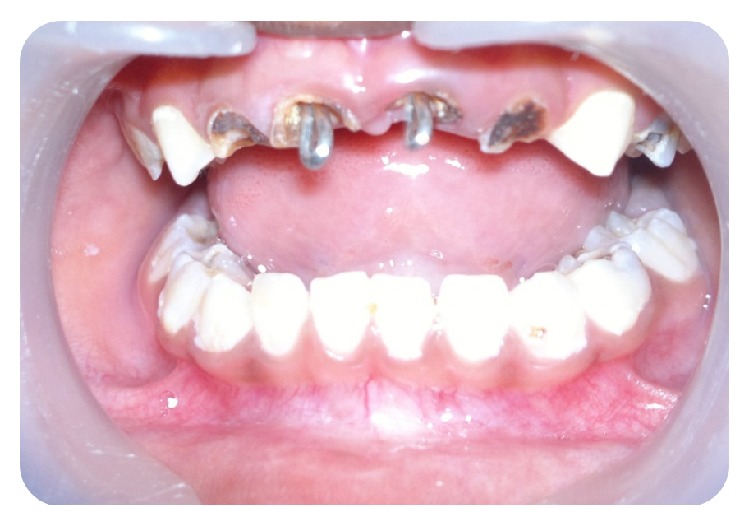
Clinical photographs with postinserted in 51, 61.

**Figure 9:**
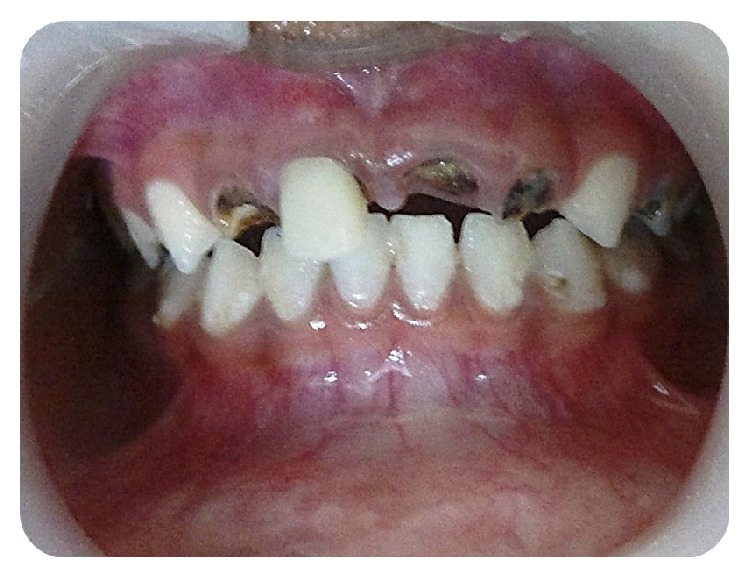
Clinical photograph of coronal build in relation to 51.

**Figure 10:**
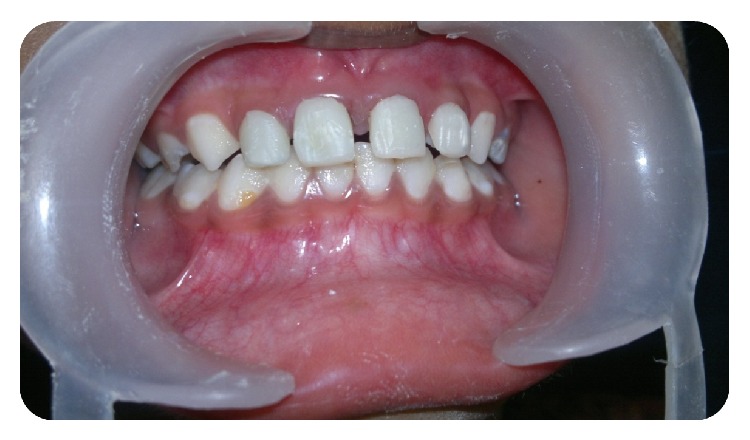
Postoperative clinical photograph.

**Figure 11:**
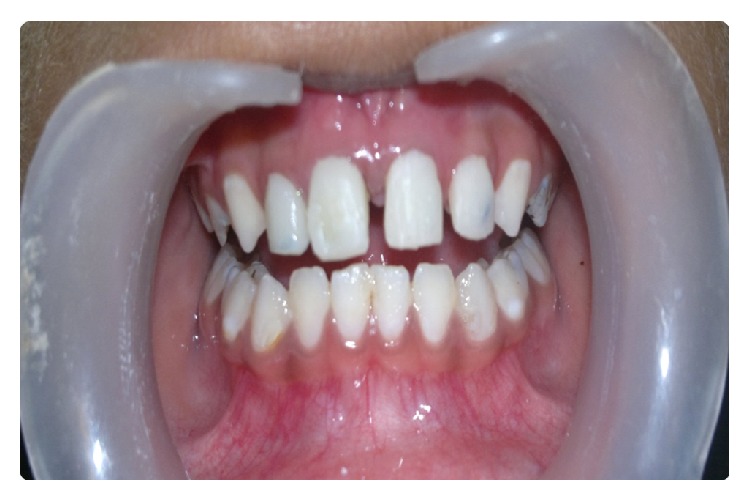
Postoperative clinical photograph after 6 months.

The post is placed in the prepared root canal and checked for adaptation (Figure 8). Mushroom shaped retention grooves are placed on the inner side of the root to create locking mechanism thereby increasing retention. Before placing the post, etching is done with phosphoric acid for 30 seconds. It is washed off and dried to reveal a frosty appearance. With the post in place, Filtek Supreme Ultra Flowable Restorative flowable composite is used to fill the root canal space. Filtek Supreme Ultra Universal Restorative nano-filled composite was used to build up the coronal part in incremental technique (Figures 9 and 10). Strip or polycarbonate crown can be used alternatively for the crown portion. The recall visits at 3 and 6 months have shown clinically satisfactory results with no mobility or fracture of the tooth (Figure 11).

## 4. Discussion

Early childhood caries is a severe form of caries occurring in children, affecting the maxillary primary incisors, leading to partial or total loss of crown structure. Loss of coronal structure compromises the longevity of the restoration, especially in the anterior primary teeth. This might cause aesthetic, speech, orthodontic, and psychological problems. Restoring severely damaged primary teeth is challenging. Hence the use of post and core or intracanal retainer is advised.

An ideal post and core should be resorbable but provide adequate retention and resistance. One of the factors governing the retention of the restoration is the adaptation of the post and core to the inner dentinal wall which is in turn governed by adhesive and cohesive forces.

The use of Omega loop as intracanal retainer was introduced by Mortada and King [10]. The adhesion between Omega wire and dentinal wall is mechanical. The wire adaptation to the internal walls is inadequate, leading to dislodgement of the wire, and radicular fracture due to excessive masticatory forces [15]. Hence retention of Omega loop is less compared to GFRC [12, 16]. GFRC provides better bonding, good strength, low risk of root fracture, good adaptation to the root canal, but the disadvantage is that it is expensive [18]. Biological posts and crowns were also tried but have a disadvantage of lack of availability from tooth bank [17, 18].

To overcome these drawbacks anchor shaped design for intracanal retention is proposed. Anchor shaped post and core have two ends—a free end and a curved end. The free end has two arms crisscrossing to the opposite side which adapt to the walls of the root. This provides extra retention by adapting to the inner wall of the root. The curved end provides strength to the coronal structure. Adaptation can be enhanced by compressing at the curved end which opens up the arms at the free end. Hence it is a simpler, easier, and inexpensive technique for treating severely damaged teeth.

## 5. Conclusion

The anchor post core design presented in this case report is an easy-to-fabricate and inexpensive alternative. The long term success of this design compared to other designs has to be investigated further. Future studies are recommended to investigate modifications in design and viability of various post and core restorations.
